# Public health opportunities and challenges in the provision of partner notification services: the New England experience

**DOI:** 10.1186/s12913-018-2890-7

**Published:** 2018-01-31

**Authors:** Sarah Magaziner, Madeline C. Montgomery, Thomas Bertrand, Daniel Daltry, Heidi Jenkins, Brenda Kendall, Lauren Molotnikov, Lindsay Pierce, Emer Smith, Lynn Sosa, Jacob J. van den Berg, Theodore Marak, Don Operario, Philip A. Chan

**Affiliations:** 1 Warren Alpert Medical School of Brown University, 222 Richmond Street, Providence, RI 02903 USA; 2 Division of Infectious Diseases, The Miriam Hospital, 164 Summit Avenue, Providence, RI 02906 USA; 3 Rhode Island Department of Health, 3 Capitol Hill, Providence, RI 02908 USA; 4 Vermont Department of Health, 108 Cherry Street, Burlington, VT 05402 USA; 5 Connecticut Department of Public Health, 410 Capitol Avenue, Hartford, CT 06134 USA; 6 Maine Center for Disease Control and Prevention, State House Station 11, Augusta, ME 04333 USA; 7 Massachusetts Department of Public Health, 250 Washington Street, Boston, MA 02108 USA; 8 New Hampshire Department of Health and Human Services, 29 Hazen Drive, Concord, NH 03301 USA; 9 Brown University School of Public Health, 121 South Main Street, Providence, RI 02903 USA

**Keywords:** STDs, HIV, Partner notification, Public health

## Abstract

**Background:**

Partner notification services (PNS) are recommended by the Centers for Disease Control and Prevention as a public health intervention for addressing the spread of HIV and other sexually transmitted diseases (STDs). Barriers and facilitators to the partner notification process from a public health perspective have not been well described.

**Methods:**

In 2015, a coalition of New England public health STD directors and investigators formed to address the increasing STD prevalence across the region (Connecticut, Maine, Massachusetts, New Hampshire, Rhode Island, and Vermont) and to promote communication between state STD programs. To evaluate barriers and facilitators of PNS programs, a survey was administered to representatives from each state to describe PNS processes and approaches.

**Results:**

Of the six PNS programs, Connecticut, Maine, Massachusetts, Vermont, and New Hampshire had combined HIV and STD PNS programs; Rhode Island’s programs were integrated but employed separate disease intervention specialists (DIS). All states performed PNS for HIV and syphilis. Maine, New Hampshire and Vermont performed services for all gonorrhea cases. Rhode Island, Connecticut, and Massachusetts performed limited partner notification for gonorrhea due to lack of resources. None of the six states routinely provided services for chlamydia, though Maine and Vermont did so for high-priority populations such as HIV co-infected or pregnant individuals. Across all programs, clients received risk reduction counseling and general STD education as a component of PNS, in addition to referrals for HIV/STD care at locations ranging from Planned Parenthood to community- or hospital-based clinics. Notable barriers to successful partner notification across all states included anonymous partners and index cases who did not feel comfortable sharing partners’ names with DIS. Other common barriers included insufficient staff, inability of DIS to identify and contact partners, and index cases declining to speak with DIS staff.

**Conclusions:**

In New England, state health departments use different strategies to implement PNS programs and referral to STD care. Despite this, similar challenges exist across settings, including difficulty with anonymous partners and limited state resources.

## Background

HIV and other sexually transmitted diseases (STDs) continue to pose a major public health burden in the United States (US). Approximately 50,000 new HIV infections occur each year, the majority among gay, bisexual and other men who have sex with men (MSM) [1]. Similarly, MSM are the leading risk group for infectious syphilis. In 2015, there were 23,872 total cases of syphilis in the US, an increase of 19% from the prior year [2]. Gonorrhea prevalence has also increased, with 395,216 cases reported in 2015, a 12.8% increase from the prior year. Prevalence of the most common reportable STD, chlamydia, also increased 5.9% during this time with over 1.5 million new diagnoses reported; this was the highest number of cases ever reported for any STD in the US. Syphilis, gonorrhea, and chlamydia also increase the risk of HIV acquisition [3].

Partner notification services (PNS) are recommended by the Centers for Disease Control and Prevention (CDC) as an effective public health intervention to reduce the transmission of STDs [4]. The CDC recommends providing PNS for all newly diagnosed or reported cases of infectious syphilis (primary, secondary, and early latent) and HIV, as well as new cases of gonorrhea and chlamydia as resources permit. PNS is the process by which individuals diagnosed with HIV and other STDs (i.e. index cases) are interviewed to collect names of sexual partners, who are subsequently informed of a possible exposure and encouraged to seek testing and care [5]. In the case of HIV, this may also include injection drug users who share needles with others. By promoting awareness, education and treatment, PNS has been shown to reduce STD transmission within sexual networks and to identify a greater number of asymptomatic infections and prevent adverse health outcomes [6, 7]. Testing partners of index cases through partner notification often results in a high yield of seropositivity for HIV and other STDs [8].

In 2015, the six New England states (Connecticut, Maine, Massachusetts, New Hampshire, Rhode Island and Vermont) formed a coalition to facilitate communication and collaboration and to address shared public health concerns such as the increasing number of new STD cases in the region. To determine current practices and barriers in the provision of PNS, each state described its PNS program including barriers and facilitators with the process. Through this state-level survey, the present study aimed to capture operational challenges and structural barriers to optimizing PNS, providing a model for other sites and laying the groundwork for future efforts to improve these programs.

## Methods

Representatives from each health department in the six New England states completed a brief online survey of PNS processes in September of 2015. The survey was formulated to investigate five general topics pertaining to how each state conducted PNS: (1) overall trends in STD epidemiology, (2) the structure of the state PNS program, (3) the number of disease intervention specialists (DIS) and their roles and responsibilities, (4) the methodologies of conducting patient interviews and notifying partners, and (5) the barriers and challenges encountered by PNS programs. A full list of questions is provided in Table 1. To examine trends in STD epidemiology, we collected publicly available data from state surveillance programs for the four most common reportable STDs (HIV, syphilis, chlamydia, and gonorrhea). The study was determined exempt from review by the Rhode Island Department of Health Institutional Review Board.

**Table 1:** Survey items evaluating PNS programs in New England

Topic	Data source
STD epidemiology	*Publicly available state surveillance data for HIV, syphilis, chlamydia and gonorrhea*
PNS program structure	1. What is the number of jurisdictions, districts or health departments in the state? 2. Do PNS protocols differ among jurisdictions? 3. In what jurisdictions are the DIS located? 4. Do you have a PNS program for HIV and/or other STDs? 5. Are partner notification services for HIV and for STDs considered part of the same program? 6. Please offer a brief description of the PNS program(s) your state provides. 7. Do PNS services cover the entire state, or just urban centers, certain health jurisdictions, or particular counties? 8. Do you have a state STD clinic? If so, how many? 9. Are these STD clinics able to bill insurance companies? 10. Do these clinics offer free testing and treatment? 11. Where do you refer people for HIV/STD testing? 12. What is the time frame for reporting infections to the department of health?
DIS officer roles	1. What additional services do the DIS provide? 2. Do DIS who participate in PNS receive specialized training in addition to the national training? If so, how are they trained? 3. How many DIS officers currently participate in PNS in total (for both HIV and other STDs)? 4. How many DIS officers currently participate in PNS for HIV? For STDs? 5. Are there any DIS officers who are based at clinics or settings other than the department of health? 6. Do the DIS work directly with surveillance staff to obtain names of index cases to contact?
PNS methodology	1. Do you provide PNS for [HIV, syphilis, gonorrhea, chlamydia]? 2. *If not*, why don’t you provide PNS for [HIV, syphilis, gonorrhea, chlamydia]? 3. *If yes*, which [HIV, syphilis, gonorrhea, chlamydia] positive index cases are contacted by PNS? 4. Do you attempt to contact all reported partners who have contact information? 5. Is there a designated/goal time to interview index cases about partners for [HIV, syphilis, gonorrhea, chlamydia]? 6. Do you employ the following notification for [HIV, syphilis, gonorrhea, chlamydia], and if so, how often: Index patient tells partner, index patient tells partner within time constraint, DIS notifies partner, dual notification, third-party notification, notification via e-mail, notification via text message, notification via hook-up apps or websites, notification via Facebook 7. What do you say in your first message to an index case? 8. How do you typically attempt to reach the partners? 9. Are contacts educated in any way about PrEP? PEP? 10. Are contacts referred in any way for PrEP? PEP?
Barriers to PNS provision	1. *For HIV, syphilis, gonorrhea and chlamydia:* What challenges do you face when it comes to successfully notifying partners and getting them tested and in care?

## Results

All six states reported having integrated PNS programs for HIV and other STDs, although in Rhode Island, these programs were integrated but not formally combined, employing separate DIS for HIV and STD partner notification. All six New England states reported implementing a uniform PNS protocol across the state. Five of the six states reported that PNS programs covered the entire state. The one exception, Rhode Island, focused partner notification efforts for gonorrhea on urban centers and on regions that collectively accounted for 75% of gonorrhea cases due to limited staff resources. Three states (New Hampshire, Vermont, and Massachusetts) reported zero state-funded STD clinics, Maine and Rhode Island reported one state-funded STD clinic each, and Connecticut reported nine STD clinics receiving some state funding.

Every state surveyed reported prioritizing PNS for newly diagnosed HIV cases and for individuals with infectious syphilis. States had between one day in Vermont and four days in Rhode Island to report newly diagnosed cases for PNS. PNS for gonorrhea was offered in Maine and Vermont, and MSM with gonorrhea were considered a priority for receiving PNS in Vermont. In New Hampshire, gonorrhea was prioritized for PNS among individuals with a diagnosis of HIV and those who had multiple gonorrhea infections in the previous 12 months. In Rhode Island, availability of PNS for gonorrhea was limited due to staff, and services were only provided in urban areas. Due to limited resources and a high number of syphilis cases, Massachusetts was also unable to offer PNS to all gonorrhea cases, though the state was working toward offering interviews to newly-infected gonorrhea patients co-infected with HIV.

Partner notification of chlamydia cases was not routinely provided in the majority of New England States. PNS for chlamydia was only offered in Vermont among pregnant women who either had untreated partners or who were untreated themselves, or who had not had a test of cure performed. In Maine, PNS for chlamydia was prioritized for people who were co-infected with HIV or gonorrhea, high-risk pregnant persons (such as those lacking prenatal care or with an untreated partner), women younger than 20 years old, men younger than 25 years old, persons who were positive for another STD in the prior six months, and STD clinic clients. Massachusetts did have PNS for chlamydia, but services were limited and available upon provider request. New Hampshire, Rhode Island, and Connecticut all cited insufficient staff and prohibitively high prevalence of chlamydia to adequately perform PNS for chlamydia. New Hampshire and Rhode Island reported insufficient funding as an additional barrier to providing PNS for chlamydia. Only Vermont made PNS available to patients with hepatitis C virus (HCV).

The number of DIS employed to conduct PNS ranged from one in New Hampshire and Vermont to 11 in Massachusetts, with considerable variation in how responsibilities were delegated. In both Maine and Rhode Island, the PNS programs was staffed by three individuals, two of whom were responsible for STD cases with a third person designated solely for HIV. In this arrangement, individuals co-infected with HIV and another STD were referred to the STD DIS in the case of a pre-existing HIV infection, and to HIV DIS if the HIV diagnosis was new. In other states, DIS shared responsibility for cases of HIV and other STDs.

Most DIS were based in centrally located state-run offices, but split their time by traveling to clinics for interviews. In all the New England states, the DIS worked directly with the surveillance staff to obtain names of newly diagnosed patients to contact. Connecticut, Massachusetts, Maine and New Hampshire provided DIS with specialized training in addition to the national CDC-sponsored training. In Connecticut, this training covered state-specific issues, phlebotomy, rapid HIV testing, confidentiality guidelines, and other topics as they arose. At the time of the survey, Massachusetts was in the process of piloting a new training program for staff, which includes in the modules “HIV Fundamentals,” “Field Safety” and “Sex in Context,” as well as those addressing other pertinent issues, along with a shadowing requirement for all new staff. New Hampshire also required that new DIS shadow trained DIS in clinics, at home visits and testing events, and during phone interviews and data entry.

In most states, DIS used phone calls as the preferred way to reach partners (Table 2). Connecticut, Massachusetts and Vermont first placed several phone calls to identified partners, and followed up with a home visit if there was no response. For HIV, syphilis, and gonorrhea, Maine DIS started with phone calls and followed up with a letter, after which a visit was made if there was no response. For chlamydia, Maine DIS relied solely on phone calls. New Hampshire DIS used phone calls to contact partners for all infections. For HIV, Rhode Island DIS first attempted a phone call, followed by a text message, a visit, a letter and an online form of contact. For syphilis, Rhode Island attempted a phone call and a text message, and for gonorrhea, DIS employed a phone call, a text, a visit, and a letter. Internet partner notification (IPN), an increasingly relevant method in light of the increased prevalence of meeting sexual partners online [9], was rarely used across the New England states. Massachusetts, Maine, and New Hampshire reported the use of smartphone applications used for meeting sexual partners (“hook-up apps”) and websites to contact partners for at least HIV and syphilis; no states reporting using Facebook to contact partners. All states reported making at least two attempts to get in touch with each partner, with the number of typical attempts varying by STD. Upon contact, DIS referred partners to a range of facilities for testing and treatment (Table 3). These included Planned Parenthood, hospital-based clinics, STD clinics, and other local providers.

**Table 2:**
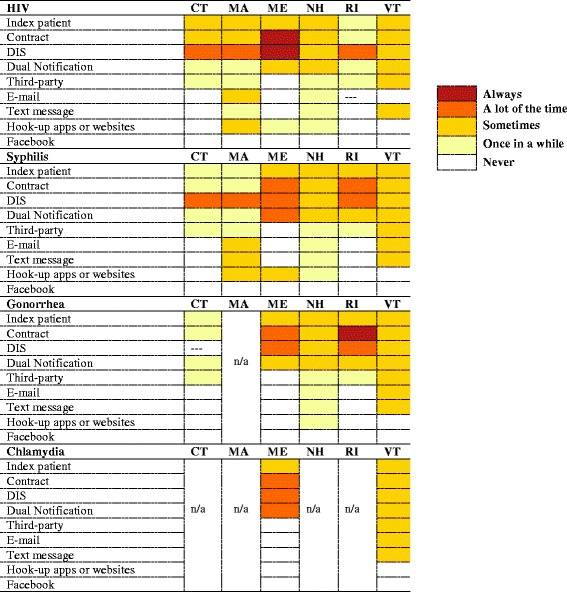
Frequency of notification method use, by infection type and state

**Table 3:** Referral sites for testing and treatment, by state

	CT	MA	ME	NH	RI	VT
Planned Parenthood	✓		✓		✓	✓
Hospital-based clinic	✓				✓	
Local providers		✓				
Local health departments	✓			✓		
Federally qualified health centers						✓
Other site		✓^a^	✓^b^	✓^b^		

^a^A network of testing and counseling sites funded by the office of HIV/AIDS (several offer free treatment, testing and counseling), relationships with other providers, and the ability to provide Bicillin for treatment of syphilis to those who do not have access

^b^Contract STD testing through ME Family Planning (Title X), which has 18 sites in central and northern Maine

^c^An additional reproductive health clinic and an additional agency for targeted HIV/HCV counseling, testing and referral services

Several challenges and barriers were reported during the partner notification process (Table 4). A primary barrier cited by survey respondents was insufficient funding for staff, which resulted in program limitations such as restricted PNS for gonorrhea and chlamydia. Additional barriers across all six states included index cases declining to speak with DIS or providing a limited number of partners, difficulty contacting partners from anonymous sexual encounters, and inability to successfully contact partners. Funding for laboratory testing was the least frequently cited barrier across infection type, followed by low utilization of PNS by referral sites.

**Table 4:** Barriers to engaging named partners for notification, by index patient infection type

	CT	MA	ME	NH	RI	VT
	HIV					
Funding for staff		✓	✓	✓	✓	
Funding for labs						✓
Low utilization by referral sites	✓	✓				✓
Index cases give anonymous partners	✓	✓	✓	✓	✓	✓
DIS are unable to contact people	✓	✓		✓	✓	
Index cases refuse to talk to the DIS	✓	✓	✓			
Index cases do not provide many names	✓	✓	✓	✓	✓	✓
Not enough staff		✓	✓	✓	✓	
Other			✓^a^			
State does not offer PNS for HIV						
	Syphilis					
Funding for staff		✓	✓	✓	✓	
Funding for labs						✓
Low utilization by referral sites	✓					✓
Index cases give anonymous partners	✓	✓	✓	✓	✓	✓
DIS are unable to contact people	✓	✓	✓	✓	✓	✓
Index cases refuse to talk to the DIS	✓	✓	✓			✓
Index cases do not provide many names	✓	✓	✓	✓	✓	✓
Not enough staff		✓	✓	✓	✓	✓
Other			✓^a^			
State does not offer PNS for syphilis						
	Gonorrhea					
Funding for staff			✓	✓	✓	✓
Funding for labs						✓
Low utilization by referral sites						✓
Index cases give anonymous partners			✓	✓	✓	✓
DIS are unable to contact people	✓		✓	✓	✓	✓
Index cases refuse to talk to the DIS	✓		✓			✓
Index cases do not provide many names	✓		✓	✓	✓	✓
Not enough staff			✓	✓	✓	✓
Other			✓^a^			
State does not offer PNS for gonorrhea		✓				
	Chlamydia					
Funding for staff			✓			✓
Funding for labs						✓
Low utilization by referral sites						✓
Index cases give anonymous partners			✓			✓
DIS are unable to contact people			✓			✓
Index cases refuse to talk to the DIS			✓			✓
Index cases do not provide many names			✓			✓
Not enough staff			✓			✓
Other			✓^a^			✓^b^
State does not offer PNS for chlamydia	✓	✓		✓	✓	

^a^Unable to use text messaging

^b^CDC does not endorse partner notification for chlamydia

## Discussion

New England represents a diverse geographical region that encompasses both rural and urban settings, with HIV and STD epidemiologic profiles mirroring the national HIV and STD trends. The present study demonstrates the opportunities and challenges to performing PNS in New England. At a time when the number of new STD cases across the US is increasing [2], PNS is an effective public health intervention for reducing HIV/STD transmission [6, 7]. All six New England states reported PNS programs implemented in accordance with current CDC guidelines [4] for both HIV and other STDs. In distinct geographic and demographic settings, each state adapted the PNS model with a variety of DIS staff and referral systems based on available resources. Programs in every state were equipped with DIS to conduct patient interviews and arrange for timely notification for new HIV and syphilis diagnoses, while providing PNS for gonorrhea and chlamydia to varying degrees. Common barriers to PNS implementation included anonymous partners, noncompliance among patients and referral sites, and insufficient staffing. Across the New England states, limited funding for staff was a consistently cited barrier to optimal PNS provision. Staffing shortages precluded most states from providing PNS for chlamydia and led to limited PNS for gonorrhea in Massachusetts, New Hampshire, and Rhode Island. Many of the states surveyed further expressed the desire to expand their PNS programs and enhance the training DIS receive. Taken together, these findings illustrate the means by which each of the New England states currently implements PNS to the extent that resources allow.

Referral methods were relatively consistent across the six states surveyed, with the most common methods consisting of in-person or telephone notification by either the index patient or the DIS. Within PNS, there are three general methods of notifying at-risk partners: *patient referral*, *provider referral*, and *contract referral* [7, 10]. Patient referral, in which health services personnel encourage patients to notify their partners on their own behalf, is the most common method of partner notification, according to surveys of patients, partners and providers [11–13]. In the case of provider referral, DIS or other third-party medical providers are responsible for contacting partners based on the information the patient provides, thus preserving patient anonymity. Contract, or conditional, referral allows the patient a specified length of time to notify partners with the understanding that the provider will notify partners who are not contacted by the established date [10]. The relative effectiveness of these strategies remains largely unknown. Studies indicate that patient referral is the most effective in eliciting partner information from patients, though it is less effective than provider or contract referral in ensuring that the partners present for medical care or are actually notified [10]. Provider referral has been described by providers as too time consuming [13] and demanding of limited resources. Patients and partners have also expressed a preference for patient notification [12]. The New England states reported using patient, contract, and provider referral methods. Provider referral was the most common referral method.

The reliance on traditional notification methods in the context of frequently reported anonymous partners and partners met online underlies a barrier faced universally by PNS in all six New England states. A 2013 Rhode Island study found that 60% of MSM newly diagnosed with HIV had used a hook-up app or website to meet sexual partners in the year prior to their diagnosis [14]. IPN has demonstrated the potential for health organizations to utilize hookup apps and websites in an effort to better engage, and reduce transmission of HIV and other STDs in this population [9, 15, 16]. IPN allows patients to anonymously inform partners of a possible exposure and aids DIS in reaching partners for whom offline contact information is unavailable [9, 16–18], and has demonstrated high acceptability among MSM in particular [19]. Despite these recent advances, the challenge of targeting anonymous partners remains one of the largest barriers to the provision of PNS. Only three states reported any use of IPN methods, despite citing challenges posed by anonymous and online partners pose in the successful application of these traditional notification methods. A movement towards IPN may substantially alleviate current difficulties reported by the New England states in effectively delivering PNS and subsequent testing and treatment to anonymous partners and partners met online.

Similar challenges exist on a global scale when it comes to identifying and treating anonymous partners and improving measurable health outcomes in resource poor settings. As partner notification becomes refined and evaluated, domestic efforts to improve notification methods can increasingly draw from innovations piloted abroad. An Australian intervention managed to increase index patients’ use of notification letters from 13% to 36% by offering information about a partner notification website alongside a positive test result [20], and research in China, the Netherlands, the United Kingdom, and elsewhere has shown that patients would be willing to use social media and the internet to better their sexual health [21, 22]. Furthermore, a systematic review and meta-analysis of global literature supported the efficacy of EPT compared to simple patient referral, indicating another area for future expansion of PNS programs [23]. As a growing body of literature on the scope of IPN has begun to emerge, from the possibility of increasing sexual health education to offering monetary compensation through IPN platforms, local health departments can begin to incorporate innovations from other jurisdictions while tailoring them to fit their specific populations and challenges.

This study has some limitations. The generalizability of the findings may be limited to the number of states surveyed. Additionally, conclusions are derived from surveys completed by a single individual, or in some cases multiple individuals, representing each state health department, precluding the consideration of alternate perspectives and experiences across those working in each department.

This survey of PNS delivery in the six New England states represents a novel undertaking in describing and comparing state-level approaches to PNS, a necessary step in subsequent efforts to enact evidence-based improvements to this critical public health intervention. As the number of new STD cases continues to rise, effective PNS is a critical public health intervention. Based on the challenges described by each state and the evolving epidemiology and social context of STD transmission, optimizing PNS merits further research to optimize outcomes.

## Abbreviations

CDC: Centers for Disease Control and Prevention
DIS: Disease intervention specialist
HCV: Hepatitis C virus
HIV: Human immunodeficiency virus
IPN: Internet partner notification
MSM: Men who have sex with men
PEP: Post-exposure prophylaxis
PNS: Partner notification services
PrEP: Pre-exposure prophylaxis
STD: Sexually transmitted disease
